# Luis Manuel Tumialán. Illustrated by Joshua Lai. Minimally Invasive Spine Surgery. A primer. 

**DOI:** 10.3325/cmj.2021.62.425

**Published:** 2021-08

**Authors:** Marina Raguž, Anđelo Kaštelančić, Darko Orešković

**Affiliations:** 1 Department of Neurosurgery, Dubrava University Hospital, Zagreb, Croatia; 2 Croatian Institute for Brain Research, Zagreb University School of Medicine, Zagreb, Croatia *marinaraguz@gmail.com*

**Field of medicine:** neurosurgery, spine surgery, orthopedics, neurology, medical education.

**Audience:** neurosurgeons, spine surgeons, orthopedic surgeons, residents, interested physicians.

**Purpose:** The book gives a well-written, comprehensive overview of minimally invasive surgery of the cervical, thoracic, and lumbar spine and provides details of various surgical techniques. History and evolution of the minimally invasive spine surgery techniques are described. Detailed surgical anatomy is presented through a number of illustrations in each chapter. The content is presented through text, intra-operative photographs, illustrations, neuroradiological images, graphs, and video content. The wealth of information provided by the book can serve as a useful source for all interested readers.

**Content:** Each of the thirteen chapters contains relevant information regarding a specific topic, accompanied by illustrations, images, and graphs, and followed by a list of references. The introductory part lists the included video content. Chapters dealing with specific operative techniques contain appropriate case illustrations, followed by a chapter conclusion.

The first chapter, *A Minimally Invasive Perspective: The Conversation,* briefly describes the premises and the main principles of learning minimally invasive spine treatment and overviews the three-dimensional spinal anatomy and essential tools such as operating table, microscope, and drill attachments. It also emphasizes the importance of the surgical team.

The second chapter, *Minimally Invasive Microdiscectomy,* gives a historical perspective of the muscle-retractor interface and the efficiency of the exposure, and describes the anatomical basis, as well as the operating room setup in detail. Special attention is given to the surgical technique; conceptualization, incision planning, exposure, bone work, division and resection of the ligamentum flavum, disc removal and closure; as well as operative time and postoperative management. Furthermore, revision microdiscectomies, complication avoidance, and repair of the cerebrospinal fluid leaks are described.

The third chapter, *Minimally Invasive Lumbar Laminectomy,* gives a historical perspective and describes patient selection, anatomical basis, and operating room setup in detail. It discusses the localization and details of the surgical technique, such as dilation and docking of the retractor, drilling, direct resection concerning canal diameter and canal depth, closure and postoperative care, long-term outcome, and complication avoidance.

The fourth chapter, *Minimally Invasive Transforaminal Lumbar Interbody Fusion,* describes various pedicle screw placements, the anatomical basis of the surgery, preoperative considerations, and operating room setup. Special attention is given to the surgical technique; the three phases of the operation are described in detail: the first phase (incision, docking, minimal access ports, and pedicle screw placement), second phase (decompression, discectomy, and end-plate preparation), and third phase (trials and interbody spacer placement); as well as postoperative management and complication avoidance.

The fifth chapter, *Minimally Invasive Far Lateral Microdiscectomy,* gives a historical perspective, and describes the anatomical basis, the anatomy of lumbar foramen, and the operating room setup. Incision planning, docking the minimal access port, bone work, pedicle, and nerve root identification, closure and postoperative care are all discussed.

The sixth chapter, *Minimally Invasive Lateral Transpsoas Interbody Lumbar Fusion,* describes advantages and disadvantages of the transpsoas approach, as well as patient selection, with special attention given to the transpsoas approach to the L IV/L V segment. Surgical anatomy is discussed in detail, with an outline of the branches of the lumbar plexus and blood vessels of the posterior abdominal wall. The chapter discusses the positioning of the patient and operating room setup, surgical planning, and the principles of electrophysiologic monitoring. Surgical technique, especially transversing the layers of the abdominal wall, orientation in the retroperitoneal space and transversing through the psoas muscle is meticulously described, followed by a description of interbody trials, device placement, fluoroscopy, potential posterior or lateral instrumentation, and closure.

The seventh chapter, *Minimally Invasive Posterior Cervical Foraminotomy,* describes the anatomical basis for minimally invasive approach, skull clamp and patient positioning, operating room setup, fluoroscopy and workflow, including incision, securing the access port, exposure using dilation, decompression in two phases (drilling the articular processes and lamina and identifying rostral and caudal pedicles), posterior cervical microdiscectomy if necessary, closure, and postoperative management.

The eighth chapter, *Minimally Invasive Posterior Cervical Laminectomy,* describes unique clinical circumstances, cervical interlaminar measurements, anatomical basis, operating room setup, skull clamp and positioning, localization, lamina exposure and drilling, hemostasis and closure.

The ninth chapter, *Anterior Cervical Discectomy with Arthroplasty or Fusion,* gives a historical perspective and describes the anatomical basis, operating room setup, patient positioning, localization, incision, exposure for single or multilevel anterior cervical discectomy and fusion, Caspar distraction posts, discectomy and osteophyte removal, spinal cord and nerve roots decompression, division and resection of the posterior longitudinal ligament, and interbody graft placement. A special part focuses on multilevel cases with instrumentation, closure, and postoperative management.

The tenth chapter, *Minimally Invasive Decompressions for Metastatic Spinal Disease,* describes the rationale for these procedures, operating room setup, patient positioning, and localization with fluoroscopy. Clinical decision making, radiological evaluation, surgical approaches and techniques with postoperative course are presented through a number of cases. In addition, minimally invasive anterior column reconstruction is presented.

The eleventh chapter, *Minimally Invasive Resection of Intradural Extramedullary Lesions within the Thoracic Spine,* gives a historical perspective and rationale for these procedures, as well as describes anatomical variations and intradural extramedullary lesion on the lumbar vs the thoracic spine. Patient positioning and localization are discussed alongside the incision planning, operative technique, including docking the minimal access port, exposure, bone work, lesion exposure, opening of the dura, resection of the lesion, dural and skin closure and postoperative care.

The twelfth chapter, *Radiation and Minimally Invasive Spine Surgery,* describes the fundamentals of fluoroscopy, origin of x-rays and components of the fluoroscope, as well as basic principles of x-ray production, emission spectrum, voltage, and current.

The thirteenth chapter, *Minimizing Ionizing Radiation in Minimally Invasive Spine Surgery,* emphasizes the principle to keep radiation as low as reasonably achievable and describes limitation of the dose, dosimetry, collimation, justification, optimization, types of fluoroscopy, acquisition time, and the digital spot technique, presenting cases managed by techniques and approaches described in the previous chapters. It also discusses the future of spinal imaging for minimally invasive spine surgery.

**Highlights:** The book contains essential information and provides an excellent overview of minimally invasive surgical procedures on the cervical, thoracic, and lumbar spine, including details of various surgical techniques accompanied by meticulous and detailed surgical anatomical illustrations. A comprehensive index at the end of the book enables quick orientation and selective reading. The text is complemented by numerous color figures, schematic presentations, neuroradiological images, and photographs, which make learning easy and exciting. Additional video content is useful for acquiring new skills, and in everyday clinical work.

**Related reading:** A number of other comprehensive medical references either in book or pocket-size handbook format are available, such as: Bridwell and DeWald's Textbook of Spinal Surgery, 4th Edition, LWW, 2019; Emory's Illustrated Tips and Tricks in Spine Surgery, 1st Edition, LWW, 2019; Pocket Atlas of Spine Surgery, 2nd Edition, Thieme, 2018; Rothman-Simeone and Herkowitz’s The Spine, 7th Edition, Elsevier, 2017; Benzel's Spine Surgery: Techniques, Complication Avoidance and Management, 4th Edition, Elsevier, 2016; Orthopedic Surgery Essentials: Spine, 2nd Edition, LWW, 2016; Minimally Invasive Spine Surgery: Advanced Surgical Techniques, 1st Edition, Jaypee Brothers Medical Pub, 2015; Handbook of Spine Surgery, 1st Edition, Thieme, 2011; Spinal Instrumentation: Surgical Techniques, 1st Edition, Thieme, 2005.

